# Tobacco use patterns in tuberculosis patients with high rates of human immunodeficiency virus co-infection in South Africa

**DOI:** 10.1186/1471-2458-13-1031

**Published:** 2013-10-31

**Authors:** Goedele MC Louwagie, Olalekan A Ayo-Yusuf

**Affiliations:** 1 School of Health Systems and Public Health, Faculty of Health Sciences, University of Pretoria, Private Bag X 323, Pretoria 0001, South Africa; 2 Department of Community Dentistry, Faculty of Health Sciences, University of Pretoria, Pretoria, South Africa

**Keywords:** Tuberculosis, Tobacco, Human immunodeficiency virus

## Abstract

**Background:**

Tuberculosis (TB) patients who smoke tobacco are at an increased risk for adverse TB treatment outcomes. This study describes tobacco use patterns among newly diagnosed TB patients, their readiness to quit, and their beliefs about tobacco-related health effects in a high HIV-burden setting in South Africa. Socio-economic and demographic factors associated with smoking were also determined.

**Methods:**

This was a cross-sectional analysis of baseline data collected for a smoking cessation study at six large tuberculosis clinics in a South African township (N = 1926). We collected information on current and past tobacco use, socio-economic and demographic status, beliefs regarding the harmful effects of smoking and quit behaviour, and motivation, using structured interviewer-administered questionnaires. TB- and HIV-related information was obtained from patient records. Data analysis entailed descriptive statistics, followed by multivariate logistic regression with backward elimination, adjusted for clustering by facility.

**Results:**

Just over one fifth of respondents (21.8%, 420/1924) reported currently smoking tobacco (males 37.6%, females 4.6%). By contrast, only 1.8% (35/1918) of all respondents reported being past smokers. Of the current smokers, about half (51.8%, 211/407) had previously attempted to quit, mainly for health reasons. The majority of respondents (89.3%, 1675/1875) believed tobacco smoking was harmful for their health and smokers were highly motivated to quit (median score 9, interquartile range 7–10). Smoking was less common among female respondents (Odds Ratio [OR] 0.10, 95% Confidence Interval [CI] 0.06-0.19) and respondents who had completed high school (OR 0.57, 95% CI 0.39-0.84), but was more common among respondents who do occasional work (OR 2.82, 95% CI 1.58-5.02), respondents who to bed hungry regularly (OR 4.19, 95% CI 2.42-7.25), those who have an alcohol problem (OR 5.79, 95% CI 3.24-10.34) and those who use illicit substances (OR 10.81, 95% CI 4.62-25.3).

**Conclusions:**

Despite documented evidence of its harmful effects, smoking is prevalent among male TB patients in this high HIV-prevalence population. Few patients have managed to quit smoking on their own. However, patients are highly motivated to stop smoking. We recommend implementing and evaluating a smoking cessation programme in tandem with TB services.

## Background

South Africa has the third highest new tuberculosis (TB) caseload in the world, and about two thirds of TB patients are also HIV-positive [1]. The outcomes of TB programmes are below the set targets, despite the widespread introduction of standardised treatment programmes and efforts to integrate HIV and TB services in the country [1]. HIV is a well-established risk factor for both high TB incidence rates and poor TB treatment outcomes, but thus far, much less attention has been paid to the role played by tobacco smoking in adversely affecting those outcomes than to the role of HIV. Several systematic reviews have found substantial evidence that tobacco smoking is associated with an increased risk of TB infection and TB disease [2-4]. There is also some evidence (albeit less robust) regarding the adverse effects of active smoking on TB mortality [2-4] and on TB outcomes in patients in whom the disease is established. Active smoking has been associated with lower treatment adherence, slower smear conversion, TB treatment failure, relapse and death during or after treatment [5-11]. Furthermore, the joint effects of smoking, TB and HIV greatly increase the risk of chronic obstructive pulmonary disease in the long term [12]. The introduction of smoking cessation services into TB programmes has therefore been advocated by several international bodies [13]. In response to this recommendation, several tobacco cessation-related studies have been undertaken in TB settings [14-16], but to our knowledge none were conducted in countries with high HIV-TB-co-infection rates. There is therefore only limited information on the patterns and prevalence of tobacco use in such settings, and on the attitudes held by TB patients about quitting. Gaining insight into these patterns and on such prevalence could help policy-makers to identify people at risk of the negative health outcomes of the triple burden of tuberculosis, HIV and tobacco use, and establish the need for cessation services as a component of TB programmes.

This study presents the baseline data of a survey undertaken among TB patients at primary care clinics in South Africa for a smoking cessation study. We describe tobacco use prevalence and patterns among this population of patients, their readiness to quit and their beliefs about the health effects of tobacco use. We also determined the socio-economic and demographic factors associated with smoking.

## Methods

### Study design and study setting

This was a cross-sectional analysis of baseline data collected from September 2011 to April 2013 in preparation for a smoking cessation study. The study took place at the six largest public service TB clinics in Soshanguve, a large urban township in the City of Tshwane Metropolitan Municipality in the Gauteng province of South Africa. The population is predominantly black Africans. TB clinics provide TB diagnosis and treatment and HIV-related care. Although TB nurses do enquire about other medical conditions and tobacco use at the time when a person is registered as a TB patient, the nurses are not required to provide structured tobacco cessation advice to patients.

### Subjects

All adult patients seeking tuberculosis treatment at the abovementioned clinics were asked to participate in the study. Children (patients under 18) and patients who had already been on treatment for over a month, very ill patients and those unable to communicate in the local language or in English were excluded from the study.

### Measurements

Information was collected on socio-economic and demographic factors, tobacco use, alcohol and illicit substance use, beliefs regarding the harmful effects of smoking and exposure to second-hand smoke (SHS). Socio-economic status was measured using questions about education, employment status, household income, an asset index (fixed telephone, mobile phone, television, radio, refrigerator and computer) and how often the respondent went to bed hungry. The “CAGE” questionnaire was used to identify respondents with possible alcohol abuse. A score of two or more on this questionnaire is indicative of an alcohol problem [17]. The CAGE questionnaire is a brief, easy-to-use, validated screening tool that is widely used in clinical settings, including in South Africa [18,19].

We inquired about both past and current tobacco smoking, smokeless tobacco (SLT) use and exposure to second-hand smoke (SHS). Past smokers were asked about past quit attempts and which quitting aids they used, if any. For current smokers, we determined the frequency and duration of tobacco use, the age at which the person started to smoke, the stage of change [20], the person’s quit attempt history, confidence and motivation to quit, and beliefs about the relationship between smoking and tuberculosis. We also inquired about quit advice received from a health care worker (HCW) at the last visit prior to this one. Questions regarding tobacco use and SLT were adapted from the Global Adult Tobacco Survey questionnaire [21]. Current smoking was defined as having smoked tobacco in the past month. We used the brief two-item validated version of the Fagerstrom Test for Nicotine Dependence (“how soon after you wake up do you smoke your first cigarette?” and “how many cigarettes do you smoke per day?”) to measure nicotine dependence [22]. Self-efficacy was measured with a slightly modified nine-item short-form self-efficacy scale derived from the original longer 31-item scale with three domains: positive affect/social situations, negative affect situations and habitual craving situations [23]. For SLT, we inquired about current and past SLT use, and type and frequency of SLT use.

TB- and HIV-related information was extracted from the individual patients’ records, which included information on the site of the tuberculosis, whether this was a first episode of TB, the patient’s HIV status and antiretroviral treatment.

Trained fieldworkers administered semi-structured questionnaires to eligible patients in the local language most commonly spoken in the study area, or in English. The study was piloted at all six clinics over a period of one month. The study was approved by the Ethics Committee of the University of Pretoria (Ethics number 116/2011).

### Data analysis and management

Data were double entered in Excel by trained full-time data capturers and exported into STATA version 12 for data comparison, cleaning and analysis [24]. Cronbach alpha internal consistency coefficients were calculated for the scales. The descriptive summary statistics consisted of percentages for categorical variables, and means with standard deviations or medians with interquartile ranges, as appropriate. The characteristics of current smokers vs. those of non-smokers were compared first, using χ^2^-tests. This analysis was followed by multivariate logistic regression with the use of backward elimination, with liberal retention criteria for non-predictive models (p < 0.2) [25]. Co-variates known to be associated with smoking from the literature review and those identified during the univariate analysis at the 25% level were included in the original multivariate model, in addition to interaction terms. Standard errors of results were adjusted for clustering by facility.

## Results

Of the 2411 patients screened, 428 (17.8%) were excluded from the interviews (247 were children, 121 were too ill or unable to understand the interview language, 49 had had more than one month of TB treatment, and 11 were excluded for other reasons). Nearly all (97.2%, 1926/1983) eligible patients gave their consent to participate in the study.

The baseline characteristics are presented in Table 1. Just over half of the patients were male (52.3%), and nearly 60% were between 18 and 39 years old. Just over a quarter of the patients (27.7%) had completed high school or had a higher qualification, and close to two-thirds had never been married (63.8%). Many respondents were poor: 79.6% lived in households with total earnings of less than ZAR 2500 per month (about 287 US dollars), 35.1% were unemployed, 30.3% lived in a dwelling with three rooms or fewer, 21.6% owned only three of the listed assets or fewer, and 11.6% went to bed hungry at least one day per month.

**Table 1:** Socio-economic, demographic and clinical characteristics of TB patients

**Characteristic**	**N**	**n (%)**
**Male**	1926	1007 (52.3)
**Age groups**	1926	
18-29		409 (21.2)
30-39		713 (37.0)
40-49		503 (26.1)
50-59		216 (11.2)
≥60		85 (4.4)
**Education**	1919	
Primary schooling or less		533 (27.8)
Some high school		854 (44.5)
Completed high school or higher qualification		532 (27.7)
**Marital status**	1921	
Now married		552 (28.7)
Divorced/separated		68 (3.5)
Never married		1225 (63.8)
Widowed		76 (4.0)
**Assets**		
≤3 assets	1917	414 (21.6)
4 assets		1209 (63.1)
5-6 assets		294 (15.3)
**Number of days hunger in past month**	1920	
0 days		1698 (88.4)
1-7 days		197 (10.3)
>7 days		25 (1.3)
**Number of rooms in household**	1918	
≤3 rooms		582 (30.3)
4 rooms		606 (31.6)
5 rooms		262 (13.7)
>5 rooms		468 (24.4)
**Employment status**	1900	
Not working*		190 (10.0)
Unemployed		666 (35.1)
Occasional work		218 (11.5)
Working full-time or part-time		826 (43.5)
**Average monthly earnings in the household**	1904	
ZAR ^&^ 1- ZAR 500		523 (27.5)
ZAR 501-ZAR 2500		992 (52.1)
>ZAR 2500		389 (20.4)
**First episode of TB**	1879	1634 (87.0)
**Pulmonary TB**	1895	1746 (92.1)
HIV-positive**	1803	1570 (87.1)
**On antiretroviral treatment (if HIV-positive)**	1570	
No		715 (45.5)
Yes		359 (22.9)
Unknown		496 (31.6)
**Alcohol problem**	1895	367 (19.4)
**Illicit drug use**	1882	77 (4.1)

*Retired/unable to work/house-maker/student/other; ^&^ZAR = South African Rand; 8.7 ZAR ≈ 1 US dollar; **HIV status unknown for 123 patients.

In terms of clinical characteristics, 87.0% of patients presented with a first episode of TB, and 92.0% had pulmonary TB. Nearly 90% of patients who knew their HIV status were HIV-positive (87.1%), but fewer than a quarter of these had records of undergoing antiretroviral treatment (22.9%) (Table 1).

Just over one fifth of patients reported current tobacco smoking (18.8% daily smokers and 3.1% occasional smokers). This figure was much higher for men (37.6%), than for women (4.6%). Very few of the total group of respondents (1.8%) were past smokers (in other words, had successfully quit smoking in the past). When limiting the analysis to non-current smokers, this figure rose marginally to 2.3% (not presented in table). The mean age of smoking initiation was 18.5 (Standard Deviation [SD] 5.6) years. Daily smokers smoked an average of 9.8 (SD 7.0) cigarettes per day. About one fifth of the daily smokers (22.7%) were nicotine-dependent (they had a two-item brief nicotine dependency score ≥4) (Table 2). Of the respondents, 4.0% confirmed currently using SLT (women 7.1%; men 1.1%). The most common form of SLT use was snuff by nose (70.2% of SLT users). Between 11% and 15% of all non-smokers reported being exposed to SHS for seven or more days per month at home, at the workplace and in a number of other settings (Table 2).

**Table 2:** Tobacco use and exposure to SHS*

**Variable**	**All patients**	**Male**	**Female**
	**n (%)**	**n (%)**	**n (%)**
	**Mean (SD)**	**Mean (SD)**	**Mean (SD)**
	**Median (IQR)**	**Median (IQR)**	**Median (IQR)**
**Current smoking**	420/1924 (21.8)	378/1005 (37.6)	42/919 (4.6)
Daily	361 (18.8)	328 (32.6)	33 (3.6)
Less than daily	59 (3.1)	50 (5.0)	9 (1.0)
**Past tobacco smoking**	35/1918 (1.8)	30/1004 (3.0)	5/914 (0.6)
**Age started smoking***(N = 327)*	18.5 (SD 5.6)	18.3 (SD 5.7)	19.7 (SD 4.8)
**Duration of smoking in years***(N = 384)*	21.6 (SD 10.7)	21.9 (SD 10.4)	19.3 (SD 13.0)
Tobacco currently smoked**			
Manufactured cigarettes	412/415 (99.3)	371/374 (99.2)	41/41 (100)
Hand-rolled cigarettes	52/413 (12.6)	48/372 (12.9)	4/41 (9.8)
Pipes	2/412 (0.5)	2/371 (0.5)	0/41 (0.0)
Cigars/cheroots/cigarillos	1/413 (0.2)	1/371 (0.3)	0/41 (0.0)
Water pipe/other	0/410 (0)	0/371 (0.0)	0/41 (0.0)
**Number of cigarettes per day***(daily smokers, N = 352)*	9.8 (SD 7.0)	9.8 (SD 7.0)	10.0 (SD 7.3)
	8 (5–12.5)	8 (5–12.0)	6.5 (4–17.5)
**Tobacco dependent**^**&**^*(daily smokers)*	74/326 (22.7)	65/297 (21.9)	9/29 (31.0)
**Current SLT**^**† **^**use**	76/1920 (4.0)	11/1005 (1.1)	65/915 (7.1)
**Uses snuff by mouth**(current SLT user)**	28/69 (40.6)	2/8 (25.0)	26/61 (42.6)
**Uses snuff by nose**(current SLT user)**	47/67 (70.2)	7/7 (100)	40/60 (66.7)
Non-smokers’ exposure to SHS smoke (≥7 days per month)**			
At home	224/1479 (15.2)	68/612 (11.1)	156/867 (18.0)
At work	170/1478 (11.5)	98/612 (16.0)	72/866 (8.3)
In café	174/1479 (11.8)	59/612 (9.6)	115/867 (13.3)
In pub	140/1479 (9.5)	57/612 (9.3)	83/867 (9.6)
In public transport	174/1479 (11.8)	52/612 (8.5)	122/867 (14.1)
In shops	169/1477 (11.4)	58/611 (9.5)	111/866 (12.8)

*SHS = second-hand smoke; **each item is a separate question, therefore patients may have responded yes to several questions; ^&^2-item brief dependency score for daily cigarette smokers ≥ 4; ^†^SLT = smokeless tobacco.

Approximately half of the current smokers (51.8%) stated that they had attempted to quit in the past 12 months (median quit duration: 21 days). However, very few patients had made use of cessation aids or services. The respondents cited their health as their main reason for wanting to quit (70.7%). Respondents appeared to be highly motivated to quit (median score 9, Interquartile Range [IQR] 7-10) and were confident that they could do so (median score 9, IQR 6–10), and 58.3% reported that they were in the preparation stage. The perceived self-efficacy score was, however, somewhat lower (median 25, IQR 17–36). Past smokers had, on average, quit about five months ago (median 5, IQR 2–11.5) and had done so mostly without the help of cessation aids (Table 3). Over two thirds of current smokers (67.3%) were asked whether they were smoking when they had last had contact with a HCW. Of these, 71.6% were advised to stop smoking completely and 13.8% to cut down on smoking (Table 3).

**Table 3:** Quit behaviour and attitudes among smokers and HCW* tobacco screening

**Variable**	**N**	**n (%)**
		**Mean (SD)**
		**Median (IQR)**
***Current smokers (N = 420)***		
**Quit attempt in the past 12 months***(all current smokers)*	407	211 (51.8)
**Duration of quit attempt in days***(smokers who attempted to quit)*	197	21 (7–60)
**Smoking cessation aids and services used**(smokers who attempted to quit)**		
Counselling	210	35 (16.7)
Nicotine replacement therapy	210	12 (5.7)
Other prescription medication	210	1 (0.5)
Traditional medicines	210	3 (1.4)
Quit line	210	1 (0.5)
Switching to smokeless tobacco	210	1 (0.5)
Other	209	2 (1.0 )
**Main reason for trying to quit in the past***(smokers who attempted to quit)*^&^	208	
Too expensive		28 (13.5)
Health reasons		147 (70.7)
Other (addictive, bad example, smell, not fair to others)		33 (15.9)
**Stage of change***(all current smokers)*	386	
Not intending to quit smoking		12 (3.1)
Considering quitting smoking in the next 6 months		149 (38.6)
Seriously thinking about quitting in the next 30 days		225 (58.3)
**Motivation score***(one item 1–10) (all current smokers)*	410	9 (7–10)
**Confidence score***(one item 1–10) (all current smokers)*	409	9 (6–10)
**Perceived self-efficacy score***(9 items, 9–45) (all current smokers)*^$^	398	25 (17–36)
**HCW assessed smoking status at last health visit**	409	275 (67.2)
**Advice received from HCW at last visit***(all patients for whom smoking status was assessed)*	275	
No advice		40 (14.6)
Advised to stop smoking completely		197 (71.6)
Advised to cut down on smoking		38 (13.8)
***Past smokers (N = 35)***		
**Age at starting tobacco smoking***(past smokers)*	22	19.4 (SD 3.8)
**Duration of smoking in years***(past smokers)*	24	17.6 (SD 10.7)
**Quit duration in months***(past smokers)*	32	5 (2–11.5)
**Smoking cessation aids and services used**(past smokers)**		
Counselling	32	3 (0.4)
Nicotine replacement therapy, other prescription medication, traditional medicine, quit line, SLT	32	0 (0.0)

*HCW = Health care worker; **each item is separate question, therefore patients may have responded yes to several questions; ^&^some column percentages may not add up to exactly 100% due to rounding off errors; ^$^Cronbach alpha for efficacy score = 0.92.

Respondents had a high awareness of the health risks of tobacco smoking. This knowledge was particularly good with regard to the risk of lung cancer (90.5%). However, knowledge regarding the risk of stroke and heart attack was lower, with only 48.5% of the patients indicating that smoking causes heart attacks and 38.2% that smoking causes strokes. Although the beliefs of smokers and non-smokers differed on the individual questions, there were no significant trends. Overall, the health risk belief score was slightly higher for smokers than for non-smokers (p = 0.036) (Table 4). Questions regarding the relationship between smoking and tuberculosis were posed only to current smokers. Of the respondents, about 40% mentioned without prompting that smokers were more likely to get TB, and a third mentioned that smoking worsens TB. These figures rose to over 80% when respondents were prompted to answer individual questions about specific adverse effects of smoking on TB (Table 5).

**Table 4:** Health beliefs of TB patients regarding the harmful effects of smoking

	**n/N (%)**	**Current smoker**	**Non-smoker**	P-value^*^
**Smoking causes serious illness**				
No	106/1875 (5.7)	30/406 (7.4)	76/1467 (5.2)	0.991
Don’t know	94/1875 (5.0)	28/406 (6.9)	66/1467 (4.5)	
Yes	1675/1875 (89.3)	348/406 (85.7)	1325/1467 (90.3)	
**Smoking causes stroke**				
No	300/1886 (15.9)	51/406 (12.6)	249/1478 (16.9)	0.281
Don’t know	866/1886 (45.9)	175/406 (43.1)	690/1478 (46.7)	
Yes	720/1886 (38.2)	180/406 (44.3)	539/1478 (36.5)	
**Smoking causes heart attack**				
No	280/1889 (14.8)	53/408 (13.0)	227/1479 (15.4)	0.679
Don’t know	692/1889 (36.6)	143/408 (35.1)	548/1479 (37.1)	
Yes	917/1889 (48.5)	212/408 (52.0)	704/1479 (47.6)	
**Smoking causes lung cancer**				
No	42/1889 (2.2)	6/408 (1.5)	36/1479 (2.4)	0.798
Don’t know	138/1889 (7.3)	41/408 (10.1)	97/1479 (6.6)	
Yes	1709/1889 (90.5)	361/408 (88.5)	1346/1479 (91.0)	
Health belief score**	7 (7–9)	8 (7–9)	7 (7–9)	0.036 ^&^

^*^Non-parametric test for trend; **3 questions about stroke, heart attack and lung cancer, score range 3–9; Cronbach alpha for health belief score = 0.60; ^&^Wilcoxon rank sum test.

**Table 5:** Perceived relationship between smoking and TB among current smokers*

	**No**	**Don’t know**	**Yes**
	**n (%)**	**n (%)**	**n (%)**
**Perceived relationship between smoking and TB (not prompted)***(N = 420)*			
A smoker is more likely to get TB	N/A**	N/A	165 (39.3)
Smoking causes TB to become worse	N/A	N/A	143 (34.1)
Smoking makes TB treatment not to work as well	N/A	N/A	94 (22.4)
Smoking causes TB to relapse	N/A	N/A	48 (11.4)
Smokers with TB are more likely to die of TB than non-smokers	N/A	N/A	34 (8.1)
Other	N/A	N/A	12 (2.9)
**Perceived relationship between smoking and TB (prompted)***(N varies between 395 and 400)*			
A smoker is more likely to get TB	29 (7.3)	29 (7.3)	340 (85.4)
Smoking causes TB to become worse	39 (9.8)	31 (7.8)	330 (82.5)
Smoking makes TB treatment not to work as well	59 (14.8)	42 (10.6)	297 (74.6)
Smoking causes TB to relapse	51 (12.9)	46 (11.7)	298 (75.4)
Smokers with TB are more likely to die of TB than non-smokers	47 (11.8)	47 (11.8)	303 (76.3)
**TB smoking belief score***(median [IQR])*^&^	15 (12–15**)**		

*Questions courtesy of Professor M. Nichter, University of Arizona; **N/A = not applicable; ^&^5 items, score range 5–15, Cronbach alpha for TB smoking belief score = 0.85.

The socio-economic and demographic factors that we found to be independently associated with smoking are presented in Table 6. Current smoking was much less common in women (OR 0.10, 95% CI 0.06-0.19), in respondents who had completed high school or had higher qualifications (OR 0.57, 95% CI 0.39-0.84), and in those living in houses with more than five rooms (OR 0.65, 95% CI 0.46-0.92). Current smoking was more common in respondents who were doing occasional work (OR 2.82, 95% CI 1.58-5.02), those who went to bed hungry for more than seven days a month (OR 4.19, 95% CI 2.42-7.25), those who had an alcohol problem (OR 5.79, 95% CI 3.24-10.34) and those who used illicit substances (OR 10.81, 95% CI 4.62-25.3). There was no interaction between sex and socio-economic indicators, sex and an alcohol problem, or drug use and an alcohol problem.

**Table 6:** Demographic and socio-economic factors associated with current smoking*

**Characteristic**	**Current smoker**	**Adjusted odds ratio**	**P-value**
	**n/N (row %)**	(95% CI)**	
**Sex**			
Male	378/1005 (37.6)	1	
Female	42/919 (4.6)	0.10 (0.06-0.19)	<0.001
**Age groups**			
18-29 years	55/409 (13.5)	1	
30-39 years	144/712 (20.2)	1.34 (0.89-2.02)	0.167
40-49 years	145/502 (28.9)	1.66 (1.04-2.65)	0.035
50-59 years	58/216 (26.9)	1.42 (0.80-2.51)	0.230
≥ 60 years	18/85 (21.2)	2.24 (1.50-3.35)	<0.001
**Education**			
Primary schooling or less	156/533 (29.3)	1	
Some high school	198/852 (23.2)	0.88 (0.59-1.32)	0.537
Completed high school or higher	61/532 (11.5)	0.57 (0.39-0.84)	0.004
**Average monthly earnings in the household**			
ZAR^#^1- ZAR500	143/522 (27.4)	1	
ZAR501- ZAR2500	207/991 (20.9)	0.89 (0.60-1.32)	0.575
>ZAR 2500	64/389 (16.5)	0.67 (0.36-1.27)	0.223 ^&^
**Employment status**			
Not working	24/190 (12.6)	1	
Unemployed	118/665 (17.7)	1.58 (0.89-2.79)	0.115
Occasional work	80/218 (36.7)	2.82 (1.58-5.02)	<0.001
Working full-time or part-time	189/825 (22.9)	1.58 (0.76-3.30)	0.220
**Assets**			
≤3 assets	116/412 (28.2)	Not retained	
4 assets	248/1209 (20.5)	in model	
5-6 assets	51/294 (17.4)		
**Number of days hunger in past month**			
0 days	351/1696 (20.7)	1	
1-7 days	54/197 (27.4)	1.18 (0.81-1.71)	0.396
>7 days	11/25 (44.0)	4.19 (2.42-7.25)	<0.001
**Number of rooms**			
≤ 3 rooms	155/582 (26.6)	1	
4 rooms	134/604 (22.2)	0.98 (0.78-1.24)	0.856
5 rooms	50/262 (19.1)	0.75 (0.44-1.28)	0.291
>5 rooms	77/468 (16.5)	0.65 (0.46-0.92)	0.014
**Alcohol problem**			
No	207/1526 (13.6)	1	
Yes	203/367 (55.3)	5.79 (3.24-10.34)	<0.001
**Drug use**			
No	347/1804 (19.2)	1	
Yes	60/76 (79.0)	10.81 (4.62-25.30)	<0.001

*Variables entered in original model: sex, age category, drug use, alcohol problem, education level, hunger, number of rooms, household income, assets, employment category, interaction sex*education, sex*household income, sex*employment category, drug use*alcohol problem, sex*alcohol problem. Pearson Goodness of fit p-value = 0.15;**Robust standard errors; ^&^p-value <0.20 in model before adjustment for clustering, therefore retained in model, ^#^ZAR = South African Rand.

## Discussion

About one in five TB patients in our study were identified as current tobacco smokers, a figure that is very close to recent survey results in 42 high-burden TB clinics in South Africa [26]. Our male and female smoking prevalence rates were also similar to those of black urban South Africans in the 2003 South African Demographic and Health Survey (SADHS) [27]. Our smoking rates were, however, much lower than those of a recent study in Cape Town, in which 56% of patients with active TB were identified as current smokers [28]. Plausible explanations for this large difference in smoking rates could be that there were more males, and more participants of ‘mixed ancestry’ in the Cape Town study, two groups which a prior study has shown to smoke more commonly [27]. HIV prevalence rates were also much higher in our study than in the Cape Town study. It may be that the effect of smoking on TB incidence is masked by the overwhelming influence of the HIV epidemic in our study setting. In addition, we may have had more under-reporting of smoking, because cotinine testing was not performed in our study, whereas it was done in the Cape Town study.

A high percentage of smokers indicated that they intended to quit. Half had already tried to quit in the past. These findings are comparable to the results of a small survey among 150 HIV-positive smokers attending HIV clinics in South Africa [29]. Similarly, 41% of TB patients in a Malaysian study had ever tried to quit and 40% were in the preparation stage of quitting [16]. However, only about 2% of all the participants in our study reported actually succeeding in quitting, a figure much lower than the 9.4% quitters in a group of black Africans in a 2002 countrywide survey [30]. The difference in quit rates suggests that the studied population might find it more difficult to quit than the general population.

Nearly 70% of the TB patients reported being screened for tobacco smoking at their last health care visit prior to this one, and 85% of the identified smokers were advised to stop smoking or to reduce their smoking. These figures were much higher than those reported on exit interviews at a community health centre in the same province in patients with a range of medical conditions: in these exit interviews, it was found that only 12.9% of patients were screened and only 11.9% of smokers were advised to quit [31]. This large difference can possibly be attributed to the fact that TB patients present with a cough, prompting HCWs to inquire about smoking. Another explanation is an increased awareness of the dangers of smoking among TB clinic nurses as a result of information received about the implementation of a tobacco cessation study at the study sites. The high percentage of patients who reported that they believe smoking is harmful for health is in keeping with high perceived current and future health risks of smoking reported in a study on HIV-positive smokers in South Africa [29].

Age, sex and marital status distribution and marital status in our respondents were similar to those reported in a recent large South African study among TB patients in South Africa [26]. Smoking was demonstrably associated with being male, having a lower level of education and lower income, which is in keeping with findings of smoking patterns in general non-TB populations in South Africa and other countries [27,30,32]. Not surprisingly, alcohol problems, substance abuse and smoking were often found to occur concurrently [33]. These findings indicate a need to address addictive behaviours simultaneously, especially because alcohol dependence may affect quit rates adversely [33,34].

Our study has a number of limitations. First, the population of TB patients is not representative of all TB patients in the province concerned (Gauteng) or the country (South Africa) and results can therefore not be generalised. Nearly one fifth of the patients were excluded because of non-eligibility, mainly because they were younger than 18 years. Other reasons included that some patients were so severely ill that they could not participate and that there were communication problems due to language differences (South Africa has 11 official languages and a number of other languages, and immigrants also attend the facilities). We also did not collect socio-demographic or smoking-related information on these patients to demonstrate comparability with study participants. This may have introduced some bias in the prevalence estimates for adults, and implies that the results cannot be applied to children. Moreover, it is possible that current smoking status was underreported, since we did not biologically validate self-reported smoking rates. Recent smoking cessation may have introduced a “sick quitter” bias, but this is unlikely to be significant, as past smoking rates were very low. As is common with questionnaires, desirable behaviours and beliefs may have been over-reported.

## Conclusions

In conclusion, our study demonstrates relatively high current smoking rates, in particular among male TB patients, despite the potential adverse effects of continued smoking on both TB- and HIV-related treatment outcomes. Many of the current smokers appeared to be highly motivated to stop smoking, and both smokers and non-smokers strongly believed smoking was harmful to their health. There was also some evidence of promising smoking screening practices among HCWs. However, the low past smoking rates in the total group of study participants are an indication that it may be difficult to succeed in quitting without assistance.

In view of these findings, we recommend the implementation and evaluation of a formal tobacco cessation programme in TB and HIV services. Smokers identified from this study were enrolled in a smoking cessation trial immediately after the administration of the baseline questionnaires. This follow-up study aims to evaluate the effectiveness of motivational interviewing by lay HCWs to assist TB patients to stop smoking tobacco. If the trial is found to be effective, lay HCWs could in future deliver smoking cessation counselling in addition to providing preventative, curative and other support functions. Such an approach would fit in with the plans to introduce community-based health outreach teams, as envisaged by the Department of Health in South Africa [35] and free up nurses’ working time to focus on the complex management of TB patients who are often dually infected with HIV.

## Abbreviations

CI: Confidence interval; HCWs: Health care workers; IQR: Interquartile range; OR: Odds ratio; SHS: Second-hand smoke; SLT: Smokeless tobacco; SADHS: South African demographic and health survey; SD: Standard deviation; TB: Tuberculosis; WHO: World Health Organisation.

## Competing interests

The authors declare no competing interests.

## Authors’ contributions

GL and OA designed and conceptualised the study. GL supervised the data collection and performed the data analysis with support from OA. GL wrote the first draft of the report. Both authors critically revised the article for intellectual content, and both have seen and approved the final draft.

## Pre-publication history

The pre-publication history for this paper can be accessed here:

http://www.biomedcentral.com/1471-2458/13/1031/prepub
